# The Diagnostic Evolution of Haematological Neoplasms: A Narrative Review of the Road to Two Genetically Focused Classification Systems Through a Resource-Limited Perspective

**DOI:** 10.3390/diagnostics16040541

**Published:** 2026-02-12

**Authors:** Caryn Benjamin, Zivanai Cuthbert Chapanduka, Nadine Rapiti

**Affiliations:** 1 National Health Laboratory Service, Department of Haematology, KwaZulu Natal 4001, South Africa; rapitin@ukzn.ac.za; 2 School of Laboratory Medicine and Medical Sciences, University of KwaZulu Natal, KwaZulu Natal 4058, South Africa; 3 National Health Laboratory Service, Department of Haematology, Cape Town 7925, South Africa; zivanai@sun.ac.za; 4 Faculty of Medicine and Health Science, Department of Pathology, Stellenbosch University, Cape Town 8000, South Africa

**Keywords:** World Health Organisation classification of Haematolymphoid Tumours fifth edition, International Consensus Classification, haematological neoplasms, molecular haematology, haematology in low and middle income countries

## Abstract

**Introduction:** Classification of haematological malignancies has evolved over centuries from multiple morphology-based classifications to a single consensus classification, the World Health Organisation (WHO) classification of tumours in 2001, which included clinical history and immunophenotype. The next two decades saw a revised WHO classification, incorporating immunophenotyping, cytogenetics, molecular genetics, morphology, and clinical features. In 2022, the WHO classification of Haematolymphoid Tumours fifth edition (WHO-HAEM5) and International Consensus Classification (ICC) integrated advanced genetic technologies. Navigating two classifications has caused uncertainty for pathologists and clinicians globally. However, there is added concern for low and middle income countries (LMICs), where diagnostic disparities compared to high income countries (HICs) already exist. The incorporation of advanced and costly genetic testing will likely widen this gap. This disparity and diagnostic evolution are the focus of this review. **Methods:** A literature search was performed for articles reporting on historical evolution of haematological malignancy diagnosis, diagnostic challenges for haematology in LMICs, haematological classification systems, overall survival, and laboratory turn-around times was performed using three scholarly databases; and a Google search was made for historic portions of this review. Ninety-two publications were included. **Results:** This narrative review describes the diagnostic and genetic evolution of haematological malignancies, and highlights disparities of laboratory diagnostics between LMICs and HICs. **Conclusions:** The existing disparities in diagnostic haematology between LMICs and HICs will likely widen due to the emphasis on advanced genetic testing in the WHO-HAEM5 and ICC. Advocacy for consistent accessibility and affordability of haematology diagnostics in LMICs is needed.

## 1. Introduction

The diagnosis and classification of haematological malignancies (HMs) have progressed significantly since 1832, when Hodgkin Disease was first described [1,2,3]. In 1845, a German doctor, Rudolf Virchow, published a description of a post-mortem sample which showed an increase in non-purulent white cells that had suppressed the red blood cells [4]. He named this disease entity ‘Leukämie’ in 1847 [5]. Thereafter, the classification of HMs progressed beyond morphology, incorporating clinical history and phenotyping into early classification models [2].

The first and second editions of the World Health Organisation (WHO) classification of tumours “blue books” were published by the WHO in the 1980s, and then by Springer, respectively [6]. The first edition contained diagnostic terms, International Classification of Diseases for Oncology morphology codes, and brief histological descriptions [6,7]. The second edition added immunohistochemistry and histological images [6,7]. In 1994, the Revised European–American Lymphoma (R.E.A.L) classification included cytogenetics and genotyping [6,7,8,9]. In the early 2000s, the International Agency for Research on cancer (IARC) invited the Society for Hematopathology (SH) and the European Association for Haematopathology (EAH) to collaborate on a multidisciplinary approach to the blue books [8,10,11]. Consequently, the 3rd edition of the WHO classification of tumours included illustrations, clinical and epidemiological information, and limited genetics [6]. The WHO 4th edition, in 2008, emphasised genetic disease drivers and highlighted tumours which could be defined by genetic aberrations [6,8,12]. In 2017, a revised 4th edition (WHO-HAEM4r) was published which included diagnostic refinements and provisional entities [6,8,13].

In 2022, the haematological community was confronted with two new classifications, the WHO Classification of Haematolymphoid Tumours 5th edition (WHO-HAEM5) and the International Consensus Classification (ICC), which are more focused on genetic aberrancies than previous classifications [10,11,14,15,16]. While the changes made are necessary for haematological diagnostics and therapeutics, they have highlighted the gap in health care between high income countries (HICs) and low and middle income countries (LMICs). This is partly because the spectrum of genetic testing required is not easily available in many LMICs [17,18,19]. Where testing is accessible, it is costly and cannot always translate into improved treatment plans due to poor access to newer drugs [17,18,19].

**Figure 1:**
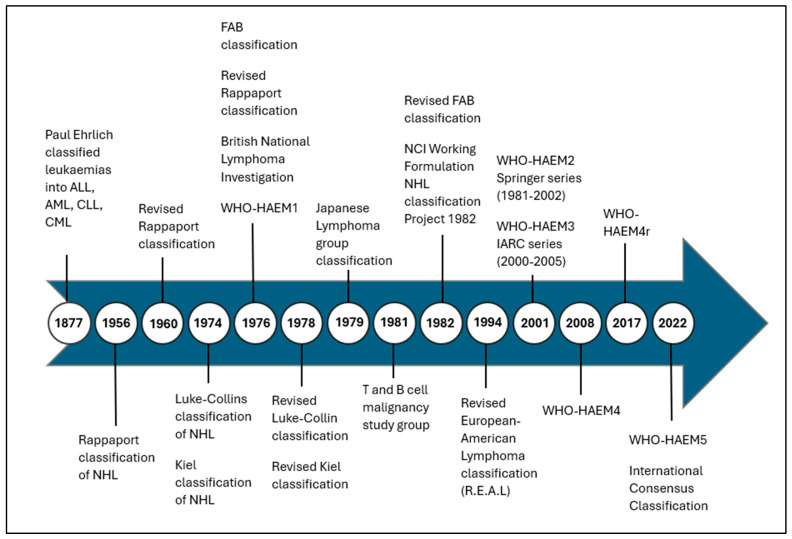
Historical evolution of Haematological Classification Systems [2,8,9,10,11,13,14,15,20,21]. Abbreviations: ALL—acute lymphoblastic leukaemia, AML—acute myeloid leukaemia, CLL—chronic lymphocytic leukaemia, CML—chronic myeloid leukaemia, NHL—Non-Hodgkin’s Lymphoma, WHO-HAEM: World Health Organisation classification of haematolymphoid tumours, FAB—French–American–British.

This article is a narrative review of the evolution (Figure 1) of the diagnosis of HMs, and a commentary on the practical application of a genetically based classification in LMICs.

## 2. Methods

A literature search was performed between October 2024 and August 2025. Articles reporting on the historical evolution of the diagnosis of HMs, diagnostic challenges for HMs in LMICs, past and current haematological classification systems, and laboratory turn-around times (TATs) were searched for using Google Scholar, Science Direct, and PubMed. Search terms used were history and diagnostic evolution of HM diagnosis, evolution of diagnostic classifications in haematology, history of the WHO classification of HM, the WHO-HAEM5 classification, ICC haematology, molecular evolution of haematology diagnosis, haematology in LMICs, disparities in global haematology, laboratory TATs for haematological diagnosis, utility of haematology guidelines in LMICs, and OS in HM.

The searches generated 20,331 articles, further refined by excluding duplicates and those older than 15 years to result in 3365. Articles including the key words but not relevant to the topic were excluded. Review articles, symposium articles, commentaries, perspective pieces, and original research from HICs and LMICs were selected. Articles focusing on interventional or clinical haematology, benign haematology, and publications on non-haematological or non-oncological diseases were excluded. For the non-historical reports, articles more than 15 years old were excluded. Based on the relevance, 92 articles were included.

## 3. Historic Evolution of Diagnostics in Haematology

### 3.1. History of Acute Leukaemias

The French–American–British (FAB) classification (1976) classified acute leukaemias (AL) based on morphology and cytochemistry, with a blast cut-off of >30% [22]. Later in 2001, the WHO classification 3rd edition of AL added immunophenotyping and cytogenetic analysis as requirements [22]. The required blast cut-off was reduced to ≥20% based on a clinical studies that showed that patients with blast counts of 20–29% were similar to those with blast counts of ≥30% [23]. The WHO 4th edition (2008) adopted distinct cytogenetic subtypes in acute lymphoid and myeloid leukaemias, and provisional subtypes with specific genetic mutations [20,22]. Although the WHO-HAEM4r (2017) described genetic and epigenetic changes which used molecular techniques for their identification, the classification remained cytogenetically based [13,24].

In the WHO-HAEM4 and 4r classifications, ALs were broadly classified into myeloid or lymphoid lineages [10,11,14,15]. They were also subclassified into those with defining genetic abnormalities, according to differentiation, or a “not otherwise specified” group [10,11,13,14,15,25]. The latest classification models, WHO-HAEM5 and ICC, have expanded the recognised genetic abnormalities which have been included as distinct entities or added to a category for genetic changes with potential for inclusion in future revisions [10,11,14,15]. New additions and alterations, which include acute myeloid leukaemia (AML) with myelodysplasia-related gene mutations, AML with myelodysplasia-related cytogenetic abnormalities, and additional recurrent genetic abnormalities, have been accepted into both the WHO-HAEM5 and ICC [10,14]. To measure residual disease in selected recurrent genetic abnormalities, quantitative molecular testing has become the standard. This requires the use of real-time quantitative polymerase chain reaction (PCR) and sequencing methods [25].

While the value of morphology and immunophenotyping is acknowledged, both the WHO-HAEM5 and ICC have adopted more genetically focused classifications and deem blast cut-offs as arbitrary in the presence of certain genetic abnormalities [10,11,14,15]. This is dependent on access to a wider range of genetic tests, including whole-genome sequencing (WGS) and whole-exome sequencing (WES).

### 3.2. History of Myelodysplastic Syndromes

In 1976, the FAB co-operative group released a publication on acute leukaemia which was based on clinical presentation, morphology and cytochemistry [26]. At this time, two broad categories of ‘dysmyelopoietic syndromes’ which could be confused with acute leukaemia, chronic myelomonocytic leukaemia (CMML) and refractory anaemia with excess blasts (RAEB), were described [26]. In 1982, the first definitive classification of myelodysplastic syndrome (MDS) proposed five forms as part of the FAB classification [27]. MDS was later included in the 2001 WHO classification based on morphology and cytogenetics [27]. The use of high-throughput techniques, WGS, WES, next-generation sequencing (NGS), and high-fidelity single nucleotide polymorphism (SNP) array in MDS has identified genetically defined disease subtypes and prognostic and risk-stratification markers [14,16,28].

Somatic mutations (SMs) have been identified in up to 89% of patients diagnosed with MDS [28]. The most common SMs are ribonucleic acid (RNA) splicing mutations in the *SF3B1* and *SRSF2* genes [28]. Both of these mutations have diagnostic predictive value, and an association with specific epigenetic mutations [28]. Mutations in the deoxyribonucleic acid (DNA) methylation protein, *TET2*, are associated with better response to hypomethylating agents such as the irreversible DNA methyltransferase inhibitors, azacitidine and decitabine, which are backbone treatments for patients with higher-risk MDS [28].

Genetically defined subclassifications of MDS are incorporated in both new classifications [10,14,16]. The diagnostic utility of SMs and karyotypic aberrancies is complicated by the entity, clonal haematopoiesis of indeterminate potential (CHIP), previously described but now accepted into both classifications [10,28].

### 3.3. History of Myeloproliferative Neoplasms

Chronic myeloid leukaemia (CML) was the first described myeloproliferative neoplasm (MPN), defined by Bennet and Virchow in 1845 [29,30,31]. Descriptions of myelofibrosis, polycythemia vera, and essential thrombocythemia followed in 1879, 1882, and 1934, respectively, and as morphological entities in the Damashek classification in 1951 [32]. Decades later, in 1960, Nowell and Hungerford discovered the Philadelphia chromosome (Ph+) [33]. Due to cytogenetic advancements, it was realised in the 1980s that the Ph+ was the result of a reciprocal translocation producing a fusion protein [33,34]. These molecular discoveries laid the foundation for the introduction of targeted therapies such as tyrosine kinase inhibitors (TKIs) in 2001, with imatinib being the pioneer drug [29,33].

The next big molecular discovery for MPNs was in 2005 with the identification of the *JAK2* V617F mutation, followed by other driver mutations, *MPL* and *CALR*, which were included as diagnostic criteria in subsequent WHO classifications [10,14,29]. While the non-*BCR::ABL1* mutations can be tested for individually using PCR, NGS is a more efficient way to screen for the driver mutation and additional aberrancies.

### 3.4. History of Myelodysplastic Syndrome/Myeloproliferative Neoplasm Overlap Syndromes

Myelodysplastic syndrome/myeloproliferative neoplasm overlap syndromes (MDS/MPNs) are a rare group of myeloid neoplasms that show features of MDS and MPNs concurrently at presentation. This group of myeloid neoplasms was first included as a distinct category in the WHO-HAEM3 in 2011 [23]. Three distinct entities, atypical CML, chronic myelomonocytic leukaemia (CMML), and juvenile myelomonocytic leukaemia (JMML), and two provisional entities, refractory anaemia with ring sideroblasts and thrombocytosis (RARS-T) and myelodysplastic syndrome/myeloproliferative neoplasm-unclassified (MDS/MPN-U), were described [23]. The next two editions saw first MDS/MPN-U and then RARS-T renamed to myelodysplastic syndrome/myeloproliferative neoplasm with ring sideroblasts and thrombocytosis (MDS/MPN-RS-T), with them accepted as distinct entities with alterations to diagnostic criteria [13,35]. The WHO-HAEM5 and ICC have both re-categorised JMML out of MDS/MPNs and into MPNs and paediatric and/or germline mutation-associated disorders [10,14]. This change was made due to the recognition that JMML is a RAS pathway activation-driven myeloproliferative neoplasm (MPN) of early childhood [10,14].

### 3.5. History of Multiple Myeloma and Other Plasma Cell Dyscrasias

In 1895, the first detailed description of plasma cells was made [36]. It was only in 2005 that a cytogenetic classification into high- and low-risk for MM was accepted [36]. Almost two decades later, the ICC have included molecular subtypes into the classification of MM [11]. These subtypes cannot be identified using cytogenetic testing, instead requiring lymphoid sequencing techniques [11]. Subtypes allow for better prognostication and treatment, especially for high-risk disease [11,37].

### 3.6. History of Lymphomas and Mature Lymphoproliferative Disorders

The first influential classification of lymphomas, in 1941–1942 by Gall and Mallory, was based on morphology, followed by the Rappaport classification in the mid-1950s [9,38]. In the 1950s to 1960s, immunological developments allowed lymphocytes to be designated as T- or B-cells, and this was incorporated into the Kiel and Luke and Collins classifications of lymphomas [9,39]. These classifications were made in relation to normal lymphoid cells of the immune system [9].

In 1970, the discordance among the four available lymphoma classifications called for a uniform classification model, known as the working formulation [9]. The R.E.A.L. classification in 1994 reported lymphoma subclassifications that corresponded with advancements in therapeutic regimens [9]. The R.E.A.L. classification also incorporated limited diagnostic translocations and markers of clonality [2]. This integrated approach continued into the WHO classifications in 2001, 2008, and 2017 [13,21].

The understanding of the genetic landscape of lymphomas has improved significantly over the last few years because of large-volume parallel sequencing [40]. Consequently, genetic testing in lymphomas has wider applicability for diagnostics, prognostics, targeted therapies, and for measurable residual disease [40].

The subclassification of diffuse large B-cell lymphoma (DLBCL) into germinal centre B- cell (GCB) or activated B-cell (ABC) types was introduced into the WHO-HAEM4r [13,16]. It now a requirement for the diagnosis of DLBCL in both the WHO-HAEM5 and ICC [11,15]. While the distinction may be made by gene expression profiling (GEP), this method is not widely available or cost effective [9]. Instead, immunohistochemical algorithms are often used as a surrogate, but they are inconsistent in their ability to yield reproducible results compatible with GEP subtype and prognostication [9,41,42,43,44,45].

Also central to subclassification of DLBCL or high-grade B-cell lymphomas (HGBCL) are rearrangements of *myc*, *bcl2*, and/or *bcl6* [9,16]. Advances in sequencing have additionally allowed for the recognition of other mutations and abnormalities which are associated with GCB or ABC cell types or site-specific DLBCLs [9,11,15]^.^

Other well described uses for molecular testing in lymphomas includes sequencing for the *MYD88* L265P mutation in lymphoplasmacytic lymphoma and the *BRAF* v600E mutation in hairy cell leukaemia [9,11]. Cytogenetic testing can detect translocations such as t(11;14) and t(14;18) in mantle cell lymphoma (MCL) and follicular lymphoma, respectively [9,11,16,40,46]. The detection of genetic abnormalities is also used for the prognostication of chronic lymphocytic leukaemia (CLL) [9,11,40,47]. CLL has seen an explosion of new targeted therapies over the past decade [40].

The WHO-HAEM5 and ICC recognise more than 30 different T-cell lymphoma subclassifications, largely based on morphology and immunophenotype [11,15,40,48,49]. However, both classifications describe two probable molecular subgroups of peripheral T-cell lymphoma (PTCL), NOS-PTCL-TBX21 (better prognosis), and PTCL-GATA3 (poor outcome) based on GEP [11,15].

With the advent of NGS lymphoid panels, the reporting and incorporation of the diagnostic and additional variants for mature B- and T-cell lymphomas has been accepted by the WHO-HAEM5 and ICC authors [11,15,40]. Unfortunately, limited accessibility of these modalities in some regions means that more conventional modalities are relied upon.

The diagnostic evolution of all major entities is summarised in Table A1.

## 4. The Molecular Evolution of Diagnostics in the Haematology Laboratory

Founding diagnostic classifications integrated clinical findings, morphology and immunophenotyping [2,22,27]. The 3rd edition of the WHO of haematolymphoid tumours incorporated cytogenetic abnormalities into the classification [20,22]. The role of genetics has increased in subsequent editions and revisions. The incorporation of WGS, WES, and NGS in research and diagnostics has led to better understanding of disease pathogenesis, diagnosis, and the use of therapeutic targets for HMs. Therefore, both new classifications allow for a diagnosis to be made with either molecular, genetic, or cytogenetic abnormalities [10,11,14,15]. Mutations driving certain entities, for example, AML, are now so well defined that the identification of some genetic abnormalities overrides previously defining disease characteristics, such as blast count [10,14].

Panel sequencing has become common practice in most laboratories where it is available. On the other hand, WGS is likely to replace conventional cytogenetic and panel sequencing in HICs because of its ability to provide quick and comprehensive genetic profiling of coding and non-coding regions [50,51,52]. In laboratories that are unable to keep up with the rapid advances in genetics, standard cytogenetic testing will likely remain the gold standard [51].

A perspective article in *Blood* from September 2022 by Cazzola and Sehn described five examples of the uses of genomic profiling used by the ICC [8]. These were the identification of driver mutations when morphology is insufficient for diagnosis, the identification of germline genetic predisposition to HM, the genetic subclassification of morphologically defined malignancy, the identification of somatic mutations for disease monitoring, and to guide prognostication and personalised therapy [8]. The genetic evolution of HM is summarised in Table A1.

## 5. What Does This Mean for Low and Middle Income Countries?

### 5.1. Global Disparities

LMICs have the most socio-economic inequality globally, including large disparities in health care between social groups [53,54]. The Lancet Commission on Diagnostics reported that approximately 47% of the global population has access to diagnostic services [55].

Although there are many international partnerships and initiatives in place to improve laboratory facilities in African and other LMICs, challenges remain [19,56]. This may be attributed to high demand, costs of maintaining equipment, limitations to specific groups, and the lack of synchronicity between outside contributors: all of which make efforts difficult to sustain [19,53,56]. In 2023, Gopal made a case for prioritising diagnostic and therapeutic health care systems in LMICs by adopting a collaborative approach to investments [57]. The need for a joint approach to strengthening pathology in LMICs is echoed in an editorial publication by Sayed, Lukande, and Fleming [58].

A publication by Ross et al. highlighted the existence of resource-limited settings (RLSs) even within HICs, which is also described by Roberts et al. [59,60]. The experience in RLSs, including rural and non-academic centres in HICs, is reported to be similar to challenges in LMICs, such as later diagnosis, limited specialised diagnostic resources and personnel, lack of health care funding, and presentation at advanced stages of disease, as well as inferior outcomes related to delayed diagnosis and limited access to medication and social determinants of health [59,61].

### 5.2. Diagnostic Challenges in LMICs

Ugwu and Nwannadi highlighted the diagnostic and management challenges for HMs in Nigeria, which are largely compatible with experiences in many LMICs [17,56,62,63,64,65,66]. Patients often present late with advanced stages and complications of the disease [56,62,64,65]. Diagnoses may be missed, incomplete, or delayed due the lack of basic ancillary testing, such as immunohistochemistry or flow cytometry (FCM) [18,54,55,56,62,64,65,66]. In low income countries (LICs), diagnosis is often made on clinical or morphological assessment alone [55,56,62]. This is far behind the progress made in some HICs, where precision diagnoses are made in shorter time frames using the latest genetic testing and patients receive therapeutic regimes strongly rooted in the latest guidelines which use the latest drugs available [57,62,66,67,68]. A case report from California by St Louis et al. described a case of acute promyelocytic leukaemia with a cytogenetically cryptic gene fusion, which was diagnosed in 48 h by rapid NGS [69]. The same authors reported the average TAT for clinical NGS testing to be 1 to 14 days [69]. A study conducted by Horgan et al. reports a range in NGS TAT from 14 days to more than 21 days in RLSs within HICs and LMICs [70].

A study by Lotodo et al. reported that some of the factors contributing to the gap between HICs and Kenya are staff shortages, inconsistent supply of reagents, and cost implications for additional testing such as FCM and molecular testing [17]. Similar findings were reported by Chamba and Mawalla on challenges faced for diagnosing lymphomas in Sub-Saharan Africa [65]. The use of ancillary diagnostic tests, which are routine in HICs, are reported to be mostly accessible to those with good health insurance in Kenya and many LMICs [17]. An Egyptian study by Rizk reported the variability in accessibility and quality between the different laboratory levels (i.e., primary health care, university, and large private laboratories) in the country [71]. This discrepancy is also the experience in Southeast Asia [72]. Often in LMICs patients are screened and provisional diagnoses are made in smaller laboratories [71]. The patients and their samples are then referred to more specialised facilities for a definitive diagnosis to be made using modalities such as FCM, cytogenetics, and molecular genetics [17,71]. This is both costly and delays diagnosis and treatment [54,71]. A similar experience was reported by authors from Mexico about diagnostic haematology in Latin America, and by Alberto and Alberto on the inequalities in access to cancer diagnostics in Southeast Asia [53,72]. A Special Issue on global haematology published by the *British Journal of Haematology* in 2017 highlighted that only a few diagnostic laboratories in LMICs have access to advanced diagnostic testing, and that this need remains unaddressed [60].

### 5.3. Cost Limitations

When advanced testing modalities are available, the controlled use of reflex diagnostic testing is limiting [17,71]. As a form of gatekeeping, some centres require tests to be requested by clinicians for the cost of the test to be paid [71]. This means that diagnoses may be incomplete and testing may be inconsistent within a laboratory [71].

### 5.4. Training and Competency

There are reported shortages of trained pathologists in certain regions of Sub-Saharan Africa [17,18,54,55,56,58,63,64,65]. Chamba reported that in 2020, Tanzania had approximately 1 pathologist for every 1 million people and 1 haematologist for every 2 million people [65]. In HICs there is one pathologist for every twenty-thousand people [64]. Where pathologists are sufficient in number, there is variable supply and quality of equipment and testing modalities available and at unaffordable rates [17,18,65]. Basic cytogenetic and molecular techniques are lacking or non-existent in some parts of Sub-Saharan Africa [18,55,64]. Similarly, opportunity and funding for training of technical laboratory staff is also deficient [17,65]. In Egypt, there are limited training avenues for laboratory technical staff, most of which are international certifications at high cost [71]. Where advanced technologies are sponsored by outside parties, the corresponding staff training and support often lags or is insufficient and requires ongoing remote support from HICs [19,65]. Local technicians, technologists, and pathologists often do not have exposure or hands-on experience with newer technologies, and require step-by-step guidance in setting up instruments and implementing new testing methods [56]. This compromises workflow and may result in diagnostic delays [19]. Compounding the shortage of trained staff is the emigration of personnel to HICs [73].

### 5.5. Implications of Diagnostic Challenges

These diagnostic challenges often play a role in morbidity and mortality rates in LMICs as timeous and accurate diagnoses are a prerequisite to planning and commencing therapy [54,67,74]. Slone et al. reported that the diagnostic and therapeutic challenges in LMICs result in a lag in paediatric oncology cure rates: ~25% in LMICs compared to ~80% in HICs [75]. Similarly, Farrag et al. demonstrated the disparity in outcomes and overall survival (OS) of childhood cancers, including ALL and AML, between Egypt and Germany [76]. Their study showed the 4-year OS for children with AML in Germany was 76% versus 0–10% in Egypt [76]. Other examples of the discrepancy in OS between HICs and LMICs are tabulated in Table 1. Rizk described one of the challenges in diagnostic haematology in Egypt to be the lack of locally developed, cost effective diagnostic guidelines [71]. International guidelines are usually developed based on cutting-edge data out of HICs [59,71,73]. These guidelines require the routine use of extensive and specialised testing panels which may not be accessible or affordable in LMICs [59,71,73]. Ross et al. reported that specially formulated limited FCM panels may be useful for the implementation and cost-effective approach of diagnostic classification guidelines, such as the WHO-HAEM5 [59]. Although essential and desirable criteria have been included in the WHO-HAEM5, designation of blast lineage is a requirement for the diagnosis of AL [59]. This requires access to immunophenotyping methods that are not readily available in LMICs [54,55,56,62,64].

### 5.6. Widening the Gap

Many African countries have struggled to keep up with providing diagnoses according to the WHO guidelines since immunophenotyping and cytogenetic studies were introduced in the WHO-HEAM 3 [17]. Subsequently, each revision has introduced more genetically defined entities, requiring increasingly advanced testing methods [10,11,13,14,15]. Unfortunately, developments in diagnostic haematology also highlights the gap between resource-rich and resource-restrained countries [17,18,86]. The widening gap is likely to make reaching recommended diagnostic standards more difficult in some LICs [65]. The development of locally derived guidelines, which take local resources and culture into account, are warranted [59,73,87].

### 5.7. Bridging the Gap

The WHO-HAEM5 has endeavoured to address the challenges faced by LMICs by creating a balance between the integration of advanced diagnostic modalities into the classification systems and what is practical on a global scale [74]. This is performed by following a hierarchical diagnostic structure for each entity, and by introducing essential and desirable diagnostic criteria [14,15,74]. While these strategies may be more applicable in LMICs, it does not remedy the gross discrepancy between practice in HICs and LMICs. Some LMICs are only just getting basic molecular and cytogenetic techniques incorporated into routine diagnostic testing [86]. In many LICs, access to these modalities is limited or unavailable [18].

A publication by Obeugu in 2025 outlined the measures taken by the WHO to build and support haematological laboratory diagnostics in Africa [56]. This includes access to FCM, molecular and cytogenetic testing, training of personnel, and working with governments to improve policy [56]. Efforts have been made to collaborate with African governments, as well as local and international organisations to narrow the gap between LMICs and HICs [56]. Even so, rural and remote areas are still not reached, and unreliable infrastructure and high costs dampen the progress made [56]. There are also several digital platforms, virtual workshops, and programmes available to pathologists in LICs, both international and in-country, which aim to improve access to teaching, support and strengthen diagnostics, and allow for easy sharing of data and knowledge [74].

The question arises whether there is value in pursuing precision diagnostics in LMICs since many have restricted and erratic access to newer therapies. In 2017, the issue of global discrepant diagnostic and therapeutic practices was highlighted by the European Union as an “unmet public health need” [74]. In 2021, the European Union established the Global Health EDCTP3 and Innovative Health Initiative Joint Undertakings, which aimed to facilitate and strengthen collaborations between public and private organisations and support research and innovation in order meet the needs of LMICs [88].

### 5.8. Challenges with Disease Monitoring

The challenges in access to genetic testing for haematological diagnosis is a well-documented problem facing pathologists and clinicians in LMICs. However, the problem does not end at diagnosis, because these testing modalities are also used for disease monitoring. Nasser and Iddy, in their publication in 2023, reported that it was common practice in Tanzania in the early 2000s for patients with CML to be monitored at their base facilities by haematological responses (HRs) only [87]. Once a patient was identified as having poor HRs to TKIs, the patient would be referred to the only registered haematology centre in Tanzania for molecular testing, serving a population of over 35 million people at the time [87,89]. Efforts have been made to close this gap by partnering with the American Society of Haematology and suppliers to introduce the GeneXpert platform for *BCR::ABL1* detection and monitoring [87]. However, due to the high-cost, patients are counselled at diagnosis that they will need to monitor *BCR::ABL1* annually or bi-annually and that the cost must be self-funded [87]. Despite the efforts made, the gap between HICs and LMICs remains large.

### 5.9. The Bottom Line

Morbidity, survival, and quality of life in patients with HMs in LMICs are known to be inferior compared to HICs [59,65,75]. Alberto and Alberto highlighted that “timely access to comprehensive and affordable cancer diagnostics improves patient survival and quality of life, ensure appropriate management decisions for optimal outcomes, and allows subsequent monitoring of cancer progression and recurrence [72].”

Despite the emphasis placed on the value of genetic studies in the diagnosis and prognostication of HMs, these modalities remain poorly accessible and unaffordable in most low and middle income regions such as Latin America, Southeast Asia, Northern Africa, and Sub-Saharan Africa [18,55,64,71,72,86].

## 6. Therapeutic Implications of Better Genetically Defined Entities

Improved molecular landscaping has opened the door to targeted and personalised therapeutic regimens [16,68,90]. The first targeted treatment for HMs was imatinib for the treatment of CML [90]. Since then numerous therapeutic targets of HMs have been identified [90]. This has resulted in development of therapies targeting key molecular role players and pathways in the neoplastic process [90]. Examples of this includes targeting the proteasome with bortezumib in myeloma, *JAK* with ruxolitinib for myeloproliferative disorders, Bruton tyrosine kinase with ibrutinib in CLL, fms-related receptor tyrosine kinase 3 (*FLT-3*) with midostaurin in AML, and *Bcl-2* with venetoclax in CLL [90].

The ongoing genetic advancements can be translated into more therapeutic options in the future to improve patient prognosis and OS, whilst limiting toxicity [90]. This goal is reliant on a diagnosis being made, centred on genetic testing.

Improved understanding of the molecular pathogenesis and drivers of haematological diseases not only allows for the development of targeted therapies but also influences changes to the classifications and diagnostic criteria of some entities. In turn, reclassifications and altered diagnostic criteria may introduce uncertainty and debate around current therapeutic regimens and obstruct certain targeted drug development and clinical trials. Kantarjian and Tefferi, in an editorial in the *American Journal of Haematology*, commented on the impact the exclusion of accelerated-phase CML from the WHO-HAEM5 will have on TKI and novel-drug development, CML treatment regimens, and consideration for allogeneic stem cell transplant [91]. Similarly, Grever et al., in a letter to the editor, described concerns about how the WHO-HAEM5 reclassification of hairy cell leukaemia variant (HCLv) into a grouped category called Splenic B-cell Lymphoma/Leukaemia with Prominent Nucleoli may hamper essential efforts toward the development of targeted therapies for HCLv [92]. It must be acknowledged that progress in the field of molecular therapeutic targeting is necessary. However, the changes made to diagnostic criteria and classification systems must strive to refine and give clarity without causing confusion and should correlate with therapeutic strategies and guidelines. As classification systems and genetic testing advance, pathology practice needs to evolve in tandem, a process that at times can be prolonged and meet resistance.

## 7. Conclusions

Great improvements have been made in understanding the pathogenesis and the diagnosis of HMs. Molecular advancements have been most remarkable over the past two decades. Thus, more accurate, genetically defined diagnoses can be made, prognostication is more clearly defined, and targeted therapeutic regimens are increasingly more available. In many cases these developments have translated into better patient outcomes. However, these benefits have not been so widely realised in LMICs and facilities outside of reference centres in HICs due to challenges in gaining access to already well-established specialised tests, newer diagnostic modalities, novel therapeutic options, and clinical trials. These disparities are a symptom of a strained and inequitable global health care system and raise concern for future sustainability of health care given the great strides being made, which come at increasing costs. The advancements are necessary at a global level; however, advocacy is urgently required for the improvement of diagnostic and therapeutic accessibility and affordability in LMICs.

## Figures and Tables

**Table 1:** Comparison of 3 to 5-year overall survival for DLBCL, BL, HL, and APL for HICs and LMICs [1,77,78,79,80,81,82,83,84,85].

Disease Entity	3- to 5-Year Overall Survival (%)	
	HICs	LMICs
*DLBCL*	60–75%	40–60%
*BL*	60–90%	40–70%
*HL*	80–>98%	65–75%
*APL*	>90%	75–78%

Abbreviations: DLBCL–diffuse large B-cell lymphoma, BL—Burkitt lymphoma, HL—Hodgkin lymphoma, APL—Acute promyelocytic leukaemia.

## Data Availability

No new data were created or analysed in this study. Data sharing is not applicable to this article.

## Abbreviations

The following abbreviations are used in this manuscript:

ABC	Activated B-cell
AML	Acute Myeloid Leukaemia
APL	Acute promyelocytic leukaemia
Bcl2	B-cell lymphoma 2
Bcl6	B-cell lymphoma 6
BCR::ABL1	Breakpoint Cluster Region–Abelson Tyrosine Kinase 1 fusion gene
BL	Burkitt Lymphoma
BRAF	B-Raf proto-oncogene
CALR	Calreticulin
CHIP	Clonal haematopoiesis of indeterminate potential
CLL	Chronic lymphocytic leukaemia
CML	Chronic myeloid leukaemia
CMML	Chronic Myelomonocytic Leukaemia
DLBCL	Diffuse Large B Cell Lymphoma
DNA	Deoxyribonucleic acid
EAH	European Association for Haematopathology
FAB	French–American–British
FCM	Flow cytometry
FISH	Fluorescent in situ hybridisation
FLT3	Fms-related receptor tyrosine kinase 3
GCB	Germinal centre B-cell
GEP	Gene expression profiling
HCLv	Hairy cell leukaemia variant
HGBCL	High Grade B-cell Lymphoma
HICs	High income countries
HL	Hodgkin lymphoma
HMs	Haematological malignancies
HR	Haematological response
IARC	International Agency for Research on cancer
ICC	International Consensus Classification
ICD-O	International Classification of Diseases for Oncology
IGHV	Immunoglobulin heavy-chain variable region gene
IPSS	International Prognostic Scoring System
JAK2	Janus kinase 2
LICs	Low income countries
LMICs	Low and middle income countries
LPD	Lymphoproliferative disorders
NGS	Next generation sequencing
MCL	Mantle cell lymphoma
MDS	Myelodysplastic neoplasms/syndromes
MM	Multiple myeloma
MPD	Myeloproliferative Disorders
MPL	Myeloproliferative Leukaemia
MPN	Myeloproliferative Neoplasms
Myc	Myelocytomatosis oncogene
MYD88	Myeloid differentiation primary response protein 88
OS	Overall survival
PCR	Polymerase chain reaction
RAEB	Refractory anaemia with excess blasts
R.E.A.L.	Revised European–American Lymphoma classification
RLSs	Resource-limited settings
RNA	Ribonucleic acid
SF3B1	Splicing factor 3b subunit 1
SH	Society for Hematopathology
SNP	Single nucleotide polymorphism
SRSF2	Serine and Arginine Rich Splicing Factor 2
TAT	Turn-around time
TET2	Tet methylcytosine dioxygenase 2
TKI	Tyrosine kinase inhibitors
TP53	Tumour protein 53
VAF	Variant allele frequency
WCT	World Health Organisation classification of Tumours
WES	Whole-exome sequencing
WGS	Whole-genome sequencing
WHO	World Health Organisation
WHO-HAEM4r	WHO classification of Haematolymphoid Tumours revised 4th edition
WHO-HAEM5	WHO Classification of Haematolymphoid Tumours 5th edition

## Appendix A

**Table A1:** Diagnostic and molecular evolution of haematological neoplasms [2,8,9,10,11,13,14,15,16,20,21,22,24,25,28,40].

Disease Entity	History of Diagnosis	Classification Systems	Molecular Evolution
	1800s: Macroscopic and microscopic assessments by Cullen		
	1970s: microscopic morphology and cytochemistry	1976: FAB 1976: WHO-HAEM1	
Acute leukaemias	2001: morphology, immunophenotyping and cytogenetic studies	2001: WHO-HAEM2 WHO-HAEM3	Conventional karyotyping
	2008: addition of defining cytogenetic and provisional molecular genetic mutations	2008: WHO-HAEM4	Conventional karyotyping FISH analysis PCR
	2017: addition of molecular genetics and sequencing techniques	2017: WHO-HAEM4r	Cytogenetics PCR Sequencing (Sanger) NGS (myeloid)
	2022: expansion on genetically defined subclassifications	2022: WHO-HAEM5 ICC	Cytogenetics PCR NGS (myeloid and lymphoid) WES/WGS
	1976: morphology, cytochemistry	1976: FAB	
	1982: morphology, cytochemistry, 5 entities	1982: revised FAB	
	2001: morphology, immunophenotyping, cytogenetics	2001: WHO-HAEM2 WHO-HAEM3	Conventional karyotyping
Myelodysplastic syndromes	2008: morphology, immunophenotyping, cytogenetics	2008: WHO-HAEM4	Conventional karyotyping, FISH analysis
	2017: morphology, immunophenotyping, cytogenetics, molecular genetics	2017: WHO-HAEM4r	Cytogenetics, PCR, Sequencing (Sanger) NGS single gene sequencing
	2022: morphology, immunophenotyping, cytogenetics, PCR, advanced sequencing techniques	2022: WHO-HAEM5 ICC	Cytogenetics SNP PCR NGS (myeloid and lymphoid) WES/WGS
	1951: clinical presentation and limited morphology	1951: Damashek classification	
	1976: morphology, cytogenetics for Ph chromosome	1976: WHO-HAEM1	Cytogenetics
	1980s–1990s: histological morphology	1980─1990: Hanover classification	
Myeloproliferative neoplasms	2001: morphology, cytogenetics, molecular genetics	2001: WHO-HAEM2 WHO-HAEM3	Conventional karyotyping FISH analysis PCR
	2008: morphology, cytogenetics, molecular genetics	2008: WHO-HAEM4	Conventional karyotyping FISH analysis PCR
	2017: morphology, cytogenetics, molecular genetics	2017: WHO-HAEM4r	Cytogenetics PCR Sequencing
	2022: morphology, cytogenetics, molecular genetics	2022: WHO-HAEM5 ICC	Cytogenetics PCR NGS
	2008: morphology, immunophenotyping, biochemical and radiological findings	2008: WHO-HAEM4	Cytogenetics +/− PCR for prognostication
Plasma cell dyscrasias	2017: morphology, immunophenotyping, biochemical and radiological findings	2017: WHO-HAEM4r	Cytogenetics +/− PCR for prognostication
	2022: morphology, immunophenotyping, biochemical and radiological findings, genetic testing	2022: WHO-HAEM5 ICC	Cytogenetics Lymphoid NGS/WES (ICC subclassification)
	Mid 1950s: morphology	1950s: Rappaport classification	
	1970s–1980s: morphology, immunophenotyping	1970s─1980s: revised Rappaport BNLI WHO-HAEM1 Luke-Collins classification Kiel Classification	
Lymphomas and mature lymphoproliferative disorders	1994: morphology, immunophenotyping, cytogenetics, molecular genetics	1994: R.E.A.L classification	FISH analysis PCR
	2001–2017: morphology, immunophenotyping, cytogenetics, molecular genetics	2001─2017: WHO-HAEM3 WHO-HAEM4 WHO-HAEM4r	Conventional karyotyping FISH analysis PCR
	2022: morphology, immunophenotyping, cytogenetics, molecular genetics	2022: WHO-HAEM5 ICC	Cytogenetics PCR NGS (lymphoid) WES/WGS
